# SARS-CoV-2 infecting endothelial cells, biochemical alterations, autopsy findings and outcomes in COVID-19, suggest role of hypoxia-inducible factor-1

**DOI:** 10.5937/jomb0-30659

**Published:** 2022-02-02

**Authors:** Vivek Ambade, Sonia Ambade

**Affiliations:** 1 Senior Faculty, Department of Biochemistry, City Pune, State Maharashtra, India; 2 H V Desai College, Department of Microbiology, City Pune, State Maharashtra, India

**Keywords:** novel coronavirus, COVID-19, SARS-CoV-2, severe acute respiratory syndrome coronavirus 2, hypoxiainducible factor-1, novi korona virus, COVID-19, SARS-CoV-2, težak akutni respiratorni sindrom koronavirusa 2, hipoinducibilni faktor-1

## Abstract

Researchers around the world have experienced the dual nature of severe acute respiratory syndrome coronavirus-2 (SARS-CoV-2), 'tragically lethal in some people while surprisingly benign in others'. There have been congregating studies of the novel coronavirus disease (COVID-19), a disease that mainly attacks the lungs but also has mystifying effects on the heart, kidneys and brain. Researchers are also gathering information to ascertain why people are dying of COVID-19, whether it is solely a respiratory disorder, a coagulation disorder or multi-organ failure. Alterations in laboratory parameters like lactate, ferritin and albumin have been established as risk factors and are associated with outcomes, yet none have not been sub stantiated with a scientific biochemical rationale. SARSCoV-2 affects the alveolar type II epithelial cells which significantly disturbs its surfactant homeostasis, deprives Na,K-ATPase of ATP, thereby disturbing the alveolar lining fluid which then gradually decreases the alveolar gaseous exchange initiating the intracellular hypoxic conditions. This activates AMP-activated kinase, which further inhibits Na,K-ATPase, which can progressively cause respiratory distress syndrome. The virus may infect endothelial cell (EC) which, being less energetic, cannot withstand the huge energy requirement towards viral replication. There - fore glycolysis, the prime energy generating pathway, must be mandatorily upregulated. This can be achieved by Hypoxia-inducible factor-1 (HIF-1). However, HIF-1 also activates transcription of von Willebrand factor, plasminogen activator inhibitor-1, and suppresses the release of thrombomodulin. This in turn sets off the coagulation cascade that can lead to in-situ pulmonary thrombosis and micro clots. The proposed HIF-1 hypothesis justifies various features, biochemical alteration, laboratory as well as autopsy findings such as respiratory distress syndrome, increased blood ferritin and lactate levels, hypoalbuminemia, endothelial invasion, in-situ pulmonary thrombosis and micro clots, and multi-organ failure in COVID-19.

## Abstract (sr)

Istraživači širom sveta iskusili su dualnu prirodu teškog akutnog sindroma koronavirusa-2 (SARS-CoV-2), koji je tragično letalan za neke ljude, a iznenađujuće benigan za druge. Brojne su studije vezano za novo korona virus oboljenje (COVID-19), koji uglavnom oštećuje pluća, ali i ima i iznenađujuće efekte na srce, bubrege i mozak. Istraživači takođe sakupljaju podatke zašto ljudi umiru od COVID-19, bez obzira da li je to samo respiratorni poremećaj, poremećaj koagulacije ili je multi-organski poremećaj. Promene laboratorijskih parametara kako što su laktat, feritin i albumin su ustanovljeni kao faktori rizika, mada još uvek nisu naučno dovoljno potvrđeni. SARS-CoV-2 deluje na alveolarne tip II epitelijelne ćelije koje značajno oštećuju površinsku homeostazu, deluju na Na,K-ATPazu, odnosno vrše oštećenje alveolarne tečnosti koja zatim postepeno umanjuje izmenu alveolarnih gasova i dovodi do izmene intracelularnih hipoksičnih uslova. Ova aktivnosti AMP-aktivirane kinaze, koja zatim inhibira Na,K,ATPazu, što zatim može progresivno da prouzrokuje respiratorni distres sindrom. Virus može da inficira endotelijalne ćelije (E]) koje postaju manje energetske, i nisu sposobne da obezbede dovoljno energetskih potreba prema viralnoj replikaciji. Prema tome, glikoliza, primarni energetski put mora da se u prvom redu reguliše. Ovo može da se postigne sa Hipoksija inducibilnim faktorom-1 (HIF-1). Međutim, HIF-1 takođe aktivira transkripciju von Willebrandovog faktora plazminogen aktivator inhibitora-1, i deluje supresivno na oslobađanje trombomodulina. Ovo nasuprot dovodi do koagulacione kaskade koja može da dovede in-situ pulmolarnu trombozu i mikro koagulaciju. Predložena HIF-1 hipoteza potvrđuje različite događaje, biohemijske promene, laboratorijske nalaze, kao i nalaze autopsije kao što su respiratorni distres sindrom, povećan nivo feritina i laktata u krvi, hipoalbuminemiju, endotelijalnu invaziju, in-situ pulmolarnu trombozu i mikro koagulaciju, kao i oštećenje brojnih organa u COVID-19.

## Introduction

The most crucial and curious question about the new coronavirus is "How deadly is it?" Majority of the new coronavirus disease-2019 (COVID-19) patients are asymptomatic [1]. Of all the COVID-19 deaths, the share of deaths in 0-17 years old is only 0.06%, while in > 65 years is 73.6% [2]. This clearly indicates that the virus is more lethal to older people. The causative agent of COVID-19, severe acute respiratory syndrome coronavirus-2 (SARS-CoV-2), thus seems to be tragically lethal in some while being surprisingly benign in others. This disease mainly attacks the lungs but also has bewildering effects on the heart, kidneys and brain. Researchers are gathering information to determine what actually kills COVID-19 patients, whether it is pneumonia, blood clots or kidney failure [3]. Presently, there is no discrete theory to explain all the features, symptoms, clinical and autopsy findings such as lethality of virus to older people, diffuse alveolar damage prevalent in younger patients, endothelial cell invasion, in-situ pulmonary thrombosis and micro clots, and multiple organ damage. Variation in laboratory parameters like lactate, ferritin and albumin have been established as risk factors and are associated with outcomes, but have not been substantiated with a scientific biochemical rationale. Here, I present the biochemical rationale and propose the Hypoxia-inducible factor-1 hypothesis to substantiate the various features, clinical laboratory as well as autopsy findings.

## Material and Methods

Only PubMed/Scopus indexed articles published on the relevant topic along with the knowledge and understanding of biochemistry, acquired over three decades, have been used for proposing the hypothesis.

## Results and Discussion

### Entry of the virus in the body

SARS-CoV-2 enters a human host cell using the angiotensin-converting enzyme 2 (ACE2) as its receptor. Recent studies have indicated 10 to 20 times higher binding affinity of SARS-CoV-2 to ACE2 than the earlier SARS-CoV [4] [5] and higher expression of ACE2 on the epithelial cells of oral mucosa [6] and nasal epithelial cells [7]. Thus, SARS-CoV-2 can replicate in these epithelial cells and can further penetrate inside the human body through these routes.

When SARS-CoV-2 enters through the mouth, it binds to and penetrates oral epithelial cells (OEC), hijacks its machinery, consumes its energy and nutrition for replication, and may even affect the gustatory system resulting in loss of taste function. Similarly, when it enters through the nose, the virus binds to and enters the nasal epithelial cells (NEC), possibly affecting the nasal olfactory epithelium and resulting in the loss of olfactory function. SARS-CoV-2 entering and damaging islets cells, resulting in acute diabetes, has been well documented [8]. Taste reduction and smell reduction has been reported in 55.4% and 41.7% respectively of 204 COVID-19 patients [9]. However, during the replication in OEC and NEC, the body gets sufficient time to tackle the virus through its own immune system. NEC also expresses the immune-associated genes [7]. The nasal cavity is in continuation with the oral cavity, which allows the virus to slowly migrate to the oropharynx, nasopharynx, throat and the upper respiratory tract (URT) and can be detected in these regions. Since no major organ or critical function is lost, the COVID-19 individual may remain asymptomatic, or may have just mild symptoms. Anosmia, with or without dysgeusia has been manifested in patients with mild to no constitutional symptoms [10] [11]. If the body's immune mechanism and lung defences are strong enough, it prevents the virus from invading the lower respiratory tract (LRT) and other organs and eventually the COVID-19 patient effectively fights off the viral invasion while being completely asymptomatic or having mild symptoms. This has been noticed in the majority of young COVID-19 individuals.

### Why is the virus so lethal to older people?

Each and every inhalation can introduce new infectious agents and irritants into the respiratory system. To protect the lungs, the conducting airways are lined with ciliated airway epithelium. Whenever microorganisms try to invade the airway epithelium, they get trapped in the mucus layer, and the cilia beat in a coordinated manner, with considerable frequency, to remove the invading pathogens. However, aging slows the ciliary beat frequency, gradually decreases the number of cilia and ciliated cells resulting in the breakdown of the lungs first and foremost line of defense [12]. In younger people, the airways are sensitive and the inhaled particles provoke vigorous coughing to expel the irritants, while in older people ( >65 years), the coughing reflex either may not be triggered, due to less sensitive sensory receptors, or may be triggered ineffectively due to reduced respiratory muscle strength [13]. In people > 65 years, the vital capacity is decreased, resulting in decreased exchange of gases, and the increased residual volume causes substantial air trapping in the lungs, making their alveolus a milieu for microbial growth. Alveoli in younger people (<65 years) have sufficient macrophages to clear the pathogens reaching alveoli, while alveoli in older people have fewer macrophages and more pro-inflammatory neutrophils that release free radicals and cytokines, which makes them more susceptible to cytokine storm that damages the alveolar structure [14]. All of these result in high risk of SARS-CoV-2 migrating to the LRT and penetrating the deep lung tissue, causing serious lung complications in the already physiologically-challenged lungs of the elderly.

### What if the SARS-CoV-2 reaches the deep lung tissue?

In lungs, the gaseous exchange is carried out in alveoli, which consists of alveolar type (AT) 1 and AT2 epithelial cells. AT1 cells, representing about 40% of the cell population but covering 90-95% of the alveolar surface area, contain scanty mitochondria and organelles. AT2 cells, representing 60% of the cell population while occupying only 5-10% of the area, are highly metabolically active with a large nucleus and their cytoplasm is rich in mitochondria, endoplasmic reticulum (ER) and prominent Golgi complex (GC) [15]. A layer of alveolar lining fluid (ALF) covers the entire surface of the alveolar epithelium, and the regulation of its volume and composition is extremely important for optimal gaseous exchange [16]. AT2 cells synthesize, assemble and regulate the secretion of functional surfactant [17]. Surfactant when released produces a monolayer over alveolar epithelium surface, which reduces alveolar surface, opsonises pathogens and facilitates their clearance. Insufficient surfactant causes alveoli collapse, pulmonary edema & respiratory distress syndrome. Nearly 10% of the secreted surfactant pool is needed to be recycled per hour as the surfactant keeps getting inactivated. Primarily, AT2 cells cause this recycling, failing which, ineffective surfactant accumulates in the alveolus causing associated complications [18]. Moreover, AT2 cells have Na,K-ATPase for the transepithelial ion transport which is crucial for the regulation of the ALF, to guarantee proper gaseous exchange [16].

In the alveolus, the AT2 cell has a high concentration of ACE2, all the infrastructure and energetics to support the replication and more importantly expression of > 20 other genes that are closely related to virus replication and transmission. This makes AT2 cells the most preferred target cells of COVID-19 in LRT [19].

### Bioenergetics burden, hypoxia, ROS, downregulation of Na,K-ATPase, breakdown of surfactant homeostasis in AT2 cells trigger lung damage

When SARS-CoV-2 enters an AT2 cell, it hijacks its entire cellular machinery and diverts its energy, essential amino acids (EAAs) and nutrition towards its own replication. If expenditure, exclusively towards the viral genome and structural proteins, are accounted for, then each virus costs a minimum of 1.7x10^7^ ATP along with a huge nutritional load of EAAs [20]. However, this does not include the expenditure towards proteases, polyproteins 1ab, 5 and 3 sequences of subgenomic mRNA, lipids, carbohydrates, transport of molecules, assembly and disassembly process in ER-GC. The huge energy expenditure and hijacking of the entire cellular machinery of the host by the virus can significantly disturb the surfactant homeostasis and deprive Na,K-ATPase of sufficient ATP, as Na,K-ATPase itself requires around 40% of cellular energy for its normal functioning [21]. Reduction in ATPase activity reduces the crucial transepithelial ion transport, which in turn disturbs the ALF resulting in decreased alveolar gaseous exchange, which is further worsened by the disturbed surfactant homeostasis. To cater for the additional energy required to support the fast viral replication, the AT2 mitochondrial electron transport chain (ETC) has to increase by consuming more glucose, fatty acids and oxygen. This increased ETC can result in more reactive oxygen species (ROS). Increased oxygen consumption with reduced oxygen delivery to the AT2 might gradually initiate intracellular hypoxic conditions in AT2. This hypoxia/ROS then activates AMP-activated kinase (AMPK) which further inhibits Na, K-ATPase. This greatly impairs lung fluid clearance [21] and can progressively increase the blood ferritin levels, as hypoxia causes more than four fold increase in ferritin content in alveolar cells. The increased blood ferritin content in COVID-19 has been abundantly reported.

Fast viral replication continues damaging the other AT2 cells exponentially. The inflammatory cytokines of endothelial cells further exacerbates the deteriorating lung. Gaseous exchange is highly reduced and the alveolus becomes fully filled with fluid, or almost air-free, and ventilators also fail to sufficiently ventilate such COVID-19 patients, leading to death by respiratory failure. Older patients with more comorbid conditions tend to die early, while younger patients with no comorbidity continue to fight for longer. The accumulation of inactive surfactant along with the cell debris, free radical damage from oxygen, cytological pleomorphic AT2 cells can cause diffuse alveolar damage, which has been reported to be more prevalent in younger patients [3].

### Respiratory failure cannot be the only cause - Endothelial cell invasion and HIF

During the clash between the immune system and SARS-CoV-2, the virus may escape into the circulatory system and spread easily because of the abundant expression of ACE2 on endothelial cells (EC) [22]. With a higher binding affinity for ACE receptors [4] [5], SARS-CoV-2 can be more potent in spreading and infecting other organs via the bloodstream [3]. The presence of viral elements within EC with evidence of EC death has also been reported [23].

The EC is not a major energy-requiring cell [24]. It has low mitochondrial content and generates more than 80% of their energy requirement through glycolysis. The oxygen consumption, which contributes to just 15% of EC energy generation, has physiologically been kept low to facilitate ECs to transfer most of the oxygen to the perivascular tissues [25]. SARS-CoV-2 infecting EC is bound to change its whole energetics, as the normally low energetic EC cannot withstand the huge energy requirement [20] towards viral replication. ECs are designed to generate energy mostly by anaerobic glycolysis, during which each glucose molecule is broken into 2 lactate molecules yielding only 2 molecules of ATP. Whereas, aerobically, each glucose molecule consuming oxygen and involving mitochondria, gets completely oxidized into 6 CO_2_molecules and generates 32 molecules of ATP. Thus, to sustain the viral replication on anaerobic glycolysis, the glycolysis must mandatorily be upregulated. This upregulation can be achieved by Hypoxia-inducible factor-1 (HIF-1), which is a transcriptional activator of genes involved in cell metabolism [26] [27]. 

HIF-1 is composed of a regulatory HIF-1α subunit and constitutively expressing HIF-1 subunit. HIF-1α subunits undergo oxygen-dependent hydroxylation by prolyl-hydroxylase domain containing protein (PHD). PHD-catalysed hydroxylation reactions require oxygen and alpha-ketoglutarate (2-oxoglutarate) as co-substrates, and iron and ascorbate as cofactors [28]. The hydroxylated HIF-1α subunits are rapidly destroyed via the ubiquitin-proteasome system (UPS) pathway. In a cellular hypoxic environment, non-hydroxylated HIF-1α subunits escape UPS degradation, and combine with HIF-1 and co-activators forming a functional HIF-1 [29]. Varieties of viral pathogens have been reported to activate the HIF-1 pathway. Hepatitis B virus, Vaccinia virus and Epstein-Barr virus can stabilize HIF-1α by interfering with prolyl hydroxylation or UPS degradation. Influenza A virus activates HIF-1 by inhibiting proteasome, that too under physiologically normal oxygen levels (normoxia), thereby mimicking a hypoxic response in normoxia [29] [30].

Thus, in COVID-19, it can be scientifically hypothesized that SARS-CoV-2 by some mechanism activates HIF-1 to manipulate the host cell environment for its own benefits. Accordingly, SARS-CoV-2 in EC activates HIF-1, which then induces the genes encoding all the glycolytic enzymes to upregulate glycolysis. Glucose transporters, phosphofructokinase-2 (generator of the most powerful glycolysis activator), phosphoglycerate kinase and pyruvate kinase, which are confirmed targets of HIF-1, are induced. HIF-1 inhibits pyruvate dehydrogenase via direct transactivation of pyruvate dehydrogenase kinase. Con sequently, pyruvate entry into tricarboxylic acid (TCA) cycle is suppressed and instead generates lactate which effluxes from the tissues via monocarboxylate transporter MCT4, which is also upregulated by HIF-1 [31]. Thus, based on this hypothesis, virus replication inside EC should cause very high consumption of glucose leading to malnutrition and high amounts of lactate in the blood. The same has been reported in 100 percent of the COVID-19 patients who died, thereby concluding that lactate levels can be used as indicators of disease progression [32]. Additionally, HIF-1 activated in EC can upregulate pro-inflammatory cytokines (IL-1, 6 and 8) and platelet-activating factor to fuel neutrophils recruitment at the site of viral invasion [33]. Increased serum IL-6 has been abundantly reported as a marker of disease progression and has been associated with fatal outcomes in COVID-19, thus favouring the proposed hypothesis.

Virally infected EC, as a damage control measure, can employ heme, as it greatly potentiates cell killing mediated by neutrophils and ROS. Consequently, EC upregulates heme oxygenase-1 (HO-1) to degrade and release Fe^2+^ from heme, and also upregulates ferritin [34]. Ferritin heavy chain (FHC) ferroxidase inactivates Fe^2+^ to Fe^3+^ inside ferritin to preclude the generation of lethal hydroxyl radical from Fe^2+^ by Fenton reaction [35]. HO-1 deficiency causing extensive EC damage amply demonstrates the significance of this defence system [34]. HIF-1 upregulates the HO-1 gene [36] and consequently necessitates more ferritin. Thus, based on the proposed HIF-1 hypothesis, one can assume that high serum ferritin reflects alveolar [37] and intense endothelial invasion by SARS-CoV-2 leading to fatal outcomes, and the same has been reported abundantly.

### HIF, coagulation abnormality and multi-organ failure

If SARS-CoV-2 indeed activates the HIF-1 pathway in EC, as hypothesized here, then it should also demonstrate its impact on procoagulant and anticoagulation behaviour of EC. Recently, it has been established that ECs produce surface regulatory proteins that prevent excessive coagulation. These include EC receptor thrombomodulin (TM), endothelial protein C receptor (EPCR), tissue factor pathway inhibitor (TFPI), and protein C (PC) [38]. Under normal physiological conditions, TM-bound thrombin converts PC that is bound to EPCR, into activated protein C (APC). For APC to be effective, its complex with protein S (PS) synthesized by EC, must be formed [39]. The resulting APC-PS complex, then inactivates the activated factor (F) VIII and FV and, therefore, limits the functions of FVIII-FIX (intrinsic tenase complex) and FX-FV (prothrombinase complexes) inhibiting coagulation under the normal physiological conditions [39]. PS is also a cofactor of TFPI [40] which inhibits the tissue factor (TF)-FVII (extrinsic tenase complex) activation of FX [38]. PS binds to activated FX and FV, inhibits activated FX independently, and downregulates thrombin generation [41]. Thus, PS has a definite function in the inhibition of coagulation. HIF-1 downregulates PS expression, resulting in its inverse relationship with PS [42]. The decreased PS might result in the inhibition of APC and TFPI. APC without its cofactor PS might fall short to prevent inactivation of activated FVIII and FV, the two cofactors essential for blood coagulation, thereby failing to prevent the FVIII-FIX intrinsic tenase and FX-FV prothrombinase complex. TFPI without the cofactor PS, may fail to inhibit TF-FVII extrinsic tenase complex activation of FX. Insufficient PS can result in an elevated amount of activated FIX, which increases the risk of venous thromboembolism. Hence, inhibition of activated FIX has been proposed as a treatment of venous thromboembolism [40]. HIF-1 activates transcription of procoagulant molecule, von Willebrand factor (VWF) [43] and induces the exocytosis of EC Weibel-Palade (WP) body (store house of P-selectin and VWF) causing release of VWF and P-selectin at the EC surface [34]. HIF-1 induces transcription and translation of the plasminogen activator (PA) inhibitor-1 [44] [45] but does not induce tissue-PA [44] and suppresses the release of TM [46]. Moreover, prostacyclin released by EC cannot exert its antiaggregatory effect on platelets as adenylyl cyclase (downstream mediator) is reduced by HIF-1. As a result, cross-linked fibrin clots are formed on the surface of endothelium and this sets off the coagulation cascade [46]. Based on the proposed hypothesis, small regions of the pulmonary capillary having SARS-CoV-2 infected ECs, can undergo coagulatory situation forming in-situ pulmonary thrombosis and micro clots. Thus in COVID-19, the pulmonary thrombotic events need not be embolic at all, instead, in-situ pulmonary thrombosis could be the culprit.

The hypothesis also explains the findings in several studies that have reported a disproportionate high number of venous clotting events as pulmonary thrombi [47] [48] without an associated increase in deep vein thrombosis (DVT) [49]. The hypothesis can also respond to the queries raised by some authors on whether the high number of pulmonary embolism (PE) are due to embolic events or are in-situ pulmonary thrombosis [49]. Thrombosis reduces the blood flow, thereby restricting the delivery of nutrients and oxygen to downstream tissues and organs. This then, gradually induces necrosis and damage to the respective organs leading to death. Multiple organ damage has been reported in COVID-19 deaths [23] [32]. Alternatively, large occlusive thrombi can detach and embolize and then occlude distal vessels resulting in thrombo-embolism. Similarly, venous thromboembolism, as a major cause of DVT and PE can be triggered [50] and the same has been reported as the cause of death [51].

## Conclusions

The proposed HIF-1 hypothesis can rationalize various features, clinical laboratory and autopsy findings such as respiratory distress syndrome, increased blood ferritin and lactate levels, hypoalbuminemia, endothelial invasion, in-situ pulmonary thrombosis and micro clots, and multi-organ failure in COVID-19.

## Dodatak

### Future plans

A definite research plan can help to prove the mechanism by which the SARS-CoV-2 activates HIF-1. In COVID-19, HIF-1 can be activated by following mechanisms:

i) Intracellular hypoxic condition.

ii) Fe^2+^ may become a limiting factor due to increased ferritin

iii) Alpha-ketoglutarate may become limiting due to its conversion to glutamate, an AA consumed towards viral structural proteins.

iv) SARS-CoV-2 component or molecule interfering in PHD-catalysed hydroxylation reac- tions or UPS degradation.

### Funding

This manuscript has not been previously published nor is not being considered for publication elsewhere.

Financial disclosure: Nothing to disclose »None declared.«

Dr. Ambade has nothing to disclose.

### Conflict of interest statement

All the authors declare that they have no conflict of interest in this work.
